# Wound Dressings Based on Chitosan-Dialdehyde Cellulose Nanocrystals-Silver Nanoparticles: Mechanical Strength, Antibacterial Activity and Cytotoxicity

**DOI:** 10.3390/polym10060673

**Published:** 2018-06-16

**Authors:** Feng Dong, Shujun Li

**Affiliations:** 1 Key Laboratory of Bio-based Material Science and Technology of Ministry of Education, Northeast Forestry University, Harbin 150040, China; lisunda@163.com; 2 Light Industry and Textile School, Qiqihar University, Qiqihar 161006, China

**Keywords:** chitosan, dialdehyde cellulose nanocrystals, silver nanoparticles, mechanical strength, antibacterial activity, cytotoxicity, wound dressings

## Abstract

The present work envisages a simple approach to synthesize a new wound dressing based on chitosan-dialdehyde cellulose nanocrystal-silver nanoparticles (CS-DCNC-AgNPs). Silver nanoparticles (AgNPs) were generated in-situ by periodate oxidation of cellulose nanocrystals to generate aldehyde functions, which were used to reduce Ag^+^ into Ag^0^ in mild alkaline conditions. Subsequently, the dialdehyde cellulose nanocrystal-silver nanoparticles (DCNC-AgNPs) were added to chitosan (CS) to form the wound dressings by solution casting method. The aim was to enhance the antibacterial effect of CS by incorporation of AgNPs and to improve the mechanical strength and hydrophobicity of CS by incorporation of DCNC that cross-linked by hydrogen bonds. The antibacterial activities were evaluated against five gram-negative bacteria, one gram-positive bacteria, and three fungi. The in vitro cytotoxicity assay was performed using the NIH3T3 cell lines by Sulforhodamine B assay. Research outputs signified that CS-DCNC-AgNPs possessed good mechanical strength and hydrophobicity, high antibacterial activity and less cytotoxicity. Our results propose that CS-DCNC-AgNPs can be a promising, safe antibacterial to be incorporated in wound dressings.

## 1. Introduction

Bacterial infections accompanying the traumatic and surgical wounds and burns have been a major threat to human health, despite decades of advances in antibiotics. Bacterial resistance to antibiotics is a big challenge due to theit irrational and excessive use [1]. Thus, researchers have been exploring novel and more efficient antibacterial dressings to reduce the risk of infections. An ideal wound dressing in the clinic can cater moist environment to the wound, endorse gaseous diffusion, prevent bacterial infection, remove excess of exudates, and can be readily removed from the wound site without causing much pain [2]. A wide array of wound dressings are available in the global market, among them, chitosan (CS) wound dressings have recently availed the attention of researchers. It is known that CS is non-toxic, biodegradable, biocompatible, and has strong antibacterial activity. Thus, it has been successfully used for the production of antibacterial dressings for biomedical applications [3]. Moreover, CS possesses good cytocompatibility, mucoadhesion, and hemostatic activity as a biomaterial, and has significant economic advantages due to its abundance [4]. CS also has certain inevitable drawbacks such as relatively poor mechanical properties [5] and its inability to eradicate bacteria at high concentration [6]. The former may be improved via a crosslinking with cellulose nanocrystals [7], and the latter by the preparation of nanocomposites that combine silver nanoparticles (AgNPs) and chitosan [8]. 

Silver ions, especially AgNPs, have notable antibacterial activity against a wide range of bacteria, yeast, fungi, and viruses due to their extremely large surface area which can provide better contact with the microorganism [9]. AgNPs are effective antibacterial agents with low toxicity to viable mammalian cells, and have been extensively studied for use in medical applications [10]. Physical, chemical, and biological methods of preparation have been used to synthesize AgNPs [11]. However, many of these methods possess some drawbacks, such as the use of toxic and harmful reductants, passivating agents, and non-aqueous solvents, which are costly, not environmentally friendly, and need special instruments for their production [12]. AgNPs also have an intensive tendency to aggregate; therefore, AgNPs need to be immobilized on suitable supports to prevent undesirable aggregation [13]. In this work, our pursuit was to use cellulose nanocrystals (CNC) as supports to build AgNPs onto the dialdehyde cellulose nanocrystals (DCNC), which is one of cellulose nanocrystal derivatives as the host for the in-situ generated AgNPs through reducing Ag^+^ into Ag^0^ in mild alkaline conditions [14]. There have been many reports about the unique properties of CNCs, such as good mechanical properties, biodegradability, and biocompatibility [15]. Singla et al. [16] reported that the CNC wound dressings could keep wounded tissue moist by controlling the wound exudates, ultimately facilitating neo-angiogenesis and re-epithelization, along with the synergistic effect of antibacterial AgNPs serving to accelerate tissue repair. 

To the best of our knowledge, no report on AgNPs loaded on DCNC (DCNC-AgNPs) as a reinforcing agent to improve the mechanical strength and antibacterial activity of CS wound dressings is available in the literature so far. In order to establish the potential applications of the prepared CS-DCNC-AgNPs films as anti-infectious wound dressings, swelling capacity, mechanical strength, and antibacterial activity were tested. Furthermore, the cytotoxicity of the CS-DCNC-AgNPs was evaluated by Sulforhodamine B (SRB) assay using NIH3T3 cell lines.

## 2. Materials and Methods 

### 2.1. Materials and Reagents 

CS (degree of deacetylation, 85%; *M*_w_, around 600 kDa), silver nitrate, sodium periodate, glycerol, acetic acid, sodium tri-polyphosphate and Sulforhodamine B were purchased from Shanghai branch of Sigma Aldrich Chemical Co., Ltd (Shanghai, China). CNC aqueous suspension (1 mg/mL) was prepared by our group according to a procedure modified from the literature [17]. All the other chemicals and reagents were of analytical grade and double distilled water was used throughout. Dulbecco's modified eagle medium (DMEM) and beef extract peptone medium used for the cultivation of bacteria were obtained from Sigma Co. Ltd. The clinical strains (C) of *Escherichia coli*, *Staphylococcus aureus*, *Klebsiella pneumoniae*, *Enterobacter cloacae*, *Streptococcus pneumoniae* and *Pseudomonas aeruginosa*, *Candida albicans*, *Candida glabrata* and *Candida krusei* were donated by the Second Affiliated Hospital of Qiqihar Medical College (Qiqihar, China). *Staphylococcus aureus* standard strain (S) (ATCC25923), *Escherichia coli* (S) (ATCC25922), *Pseudomonas aeruginosa* (S) (ATCC27853)and NIH3T3 cell lines were obtained from Laboratory of Biochemistry of Qiqihar University (Qiqihar, China).

### 2.2. Synthesis of DCNC-AgNPs

One gram of NaIO_4_ was added to 100 mL of CNC suspension (1 mg/mL). After stirring for 24 h at room temperature in dark conditions, the dialyzed solution was collected to give the DCNC. Five milliliters of silver ammonia solution (0.1 mg/mL) was added to 5 mL of DCNC (1 mg/mL), and the mixture was heated at 50 °C for 30 min. The resulting brown suspension was signed DCNC-AgNPs.

### 2.3. Synthesis of CS-DCNC-AgNPs Wound Dressings

The preparation of CS- DCNC-AgNPs film forming solutions was in accordance with our previous methods [18]. The chitosan solution was prepared by mixing 1 g of CS, 2 mL of acetic acid and 98 mL of distilled water. Subsequently, a desired amount of DCNC-AgNPs suspension (0.2 mg/mL) was added into the CS solution containing 0%, 3%, 5%, and 10% DCNC-AgNPs, that were designated as CS, CS-DCNC-AgNPs (3%), CS-DCNC-AgNPs (5%) and CS-DCNC-AgNPs (10%), respectively. The film forming solutions were cast on a plastic mold and left at 25 ± 2 °C for 24 h, until complete evaporation of water had occurred. The spreaded films were stored in a desiccator containing saturated magnesium nitrate solution at 25 °C and 50% of relative humidity for 48 h before testing. 

### 2.4. Characterization of DCNC-AgNPs and CS-DCNC-AgNPs

UV-Vis absorption spectra of DCNC-AgNPs solution were measured by UV-2450 spectrophotometer (Shimadzu, Kyoto, Japan). The produced DCNC-AgNPs were imaged using transmission electron microscope (TEM, Hitachi 7560, Tokyo, Japan) at 200 KV by placing 5 μL of DCNC-AgNPs solution on a carbon coated copper grid and dried at room temperature. The X-ray photoelectron spectroscopy (XPS) analysis of CS-DCNC-AgNPs film samples was examined by ESCALAB 250Xi (Thermo, Waltham, MA, USA). The CS-DCNC-AgNPs film samples were analyzed on a Fourier transform infrared spectrometer (FTIR, Nicolet magna 560, Madison, AL, USA) in the range of 400–4000 cm^−1^ at a resolution of 4 cm^−1^ and 32 scans. CS-DCNC-AgNPs film samples were cut into a 4 mm × 4 mm piece, frozen in liquid nitrogen, and sputtered with gold. Surface microstructure was examined by scanning electron microscope (SEM, Quanta 200, Eindhoven, The Netherlands) at the accelerating voltage of 20 KV. 

### 2.5. Mechanical Strength Study

Tensile strength (TS), tensile modulus (TM) and elongation at break (Eb) of CS-DCNC-AgNPs films were measured by using Universal Testing Machine (Model H5KT, Tinius-Olsen Inc., Horsham, PA, USA) according to Huq et al. [19]. The dimensions of sample were 60 mm × 15 mm × 0.03 mm and the gauge length was 10 mm. The crosshead speed was set at 5 mm/min. Five specimens were tested for each sample.

### 2.6. Swelling Ratio Study

The swelling ratio test was analyzed for 24 h. The CS-DCNC-AgNPs films were dried at 37 °C incubator before cut into 1 cm × 1 cm cube. The dried films were then weighed (*W_d_*) before being immersed in water for 1–24 h. The wet weight of the films (*W_t_*) was measured by taking out the films from water and blotting with a filter paper to remove the surface adsorbed liquid and weighing the films. Five specimens were tested for each sample. The swelling ratio was calculated by the following Equation [20]:

$$\mathrm{Swelling}\;\mathrm{ratio}=[\frac{W_{t}-W_{d}}{W_{d}}]\times 100\%$$

### 2.7. Antibacterial Activity Study

The antibacterial activity of CS-DCNC-AgNPs films was tested by the disc diffusion method, as described by Arjunan et al. [21] with slight modifications. For antibacterial assessment of the CS-DCNC-AgNPs, *Staphylococcus aureus* clinical strain and standard strain was selected as the representatives of Gram-positive bacteria. *Escherichia coli*, *Klebsiella pneumoniae*, *Enterobacter cloacae*, *Streptococcus pneumoniae* and *Pseudomonas aeruginosa* clinical strain, and *Escherichia coli* and *Pseudomonas aeruginosa* standard strain were selected as representatives of Gram-negative bacteria. Optical density was used as an estimation of the colony forming units (CFU) in the suspension. Preliminary experiments verified that by using this protocol, the bacteria loaded were in the log phase. Isolated colonies of the test strains were cultured in a nutrient broth overnight at 100 rpm and 37 °C in order to obtain a fresh bacteria suspension suitable for inoculation. Subsequently, 100 μL of freshly grown bacteria were inoculated (10^7^ CFUs mL^−1^) for all the strains on beef extract peptone medium, and the plates were incubated at 37 °C for 24 h. A similar procedure was also followed for the *Candida albicans*, *Candida glabrata*, and *Candida krusei* clinical strains. One hundred microliters of freshly grown fungi (10^6^ CFUs mL^-1^) for all the strains was inoculated on the DMEM medium, and the plates were incubated at 35 °C for 24 h. The filter papers with CS-DCNC-AgNPs film forming solutions were sterilized using UV light for 30 min, and then placed on the plates and incubated for 24 h at 37 °C for antibacterial activity assay, and 30 °C for antifungal activity assay. The diameter of inhibition zone was measured in mm. Pure CS film was used for the comparative study. The specimens were tested in triplicate. 

### 2.8. Cytotoxicity Study by SRB 

The cytotoxicity assay of CS-DCNC-AgNPs was tested by the SRB assay as described by Doshi et al. [22], with some modifications. Based on our preliminary experiments, 0.2 g CS-DCNC-AgNPs film forming solutions was added to 1mL DMEM containing 10% fetal bovine serum at 37 °C for 24 h. The extract obtained was filtered with a 0.22 μm membrane filter for the removal of bacteria. Then, the sample extract (200 mg/mL) was diluted to 200, 40, 20, 10, 4, and 2 mg/mL with culture medium, according to our preliminary experiment, and the diluted sample extracts were designated as D200, D40, D20, D10, D4, and D2, respectively. As a control, the culture media with no sample extract was designated as D0.

NIH3T3 cells were seeded in a 96-well plate at a density of 4 × 10^3^ cells/well and incubated for 24 h at 37 °C under a humidified atmosphere of 95% relative humidity and 5% CO_2_ atmosphere. After 24 h incubation, the culture medium was removed and replaced by the diluted sample extract [23]. Six specimens were tested for each sample. 

At the end of incubation, SRB solution (50 μL) at 0.4% (*w*/*v*) in 1% acetic acid was added to each of the wells, and the plates were incubated for 10 minutes at room temperature. Subsequently, the residual dye was removed by acetic acid. The plates were air-dried. The bound stain was eluted with 100 μL trizma base, and the absorbance was read at 492nm using a microplate reader (Sunrise-basic, Tecan, Austria) [24]. Finally, the relative growth rate (RGR) was calculated by the following equation:

$$\mathrm{RGR}\;\%=[\frac{\mathrm{Absorbance}\;\mathrm{of}\;\mathrm{the}\;\mathrm{test}\;\mathrm{sample}}{\mathrm{Absorbance}\;\mathrm{of}\;\mathrm{the}\;\mathrm{control}}]\times 100$$

The toxicity levels of the samples and the safety standards were determined as described by Liu et al. [25]: toxicity level 0 (RGR > 100%, safe), toxicity level I (RGR = 75–100%, safe), toxicity level II (RGR = 50–75%, insecurity), toxicity level III (RGR = 25–50%, insecurity), toxicity level IV (RGR = 1–25%, insecurity) and toxicity level V (RGR < 1%, insecurity). 

### 2.9. Statistical Analysis

The results were expressed as the means ± standard deviations. All experimental data were compared using the AVOVA with Tukey post hoc test, and statically significant values were denoted by * (*p* < 0.05) and ** (*p* < 0.01). 

## 3. Results

### 3.1. Characterization of DCNC-AgNPs and CS-DCNC-AgNPs

**Figure 1:**
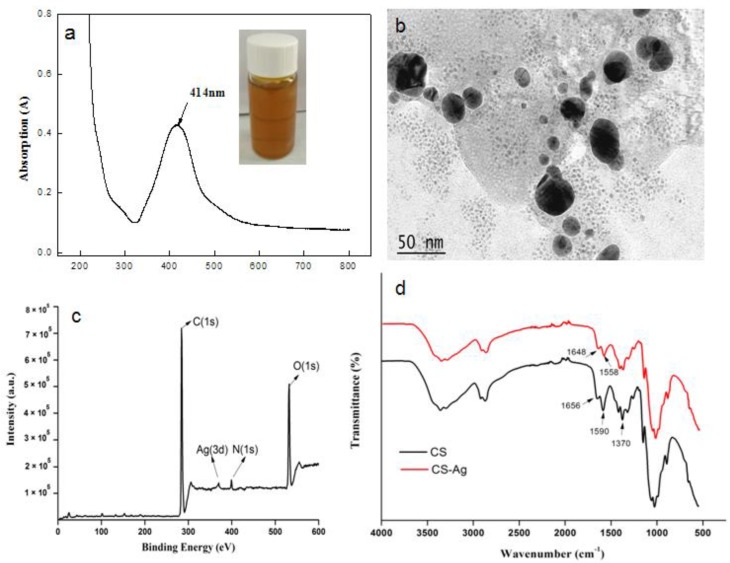
Characterization of DCNC-AgNPs and CS- DCNC-AgNPs: (**a**) UV-Vis absorption spectra of DCNC-AgNPs. The figure in the inset shows the color of synthesized DCNC-AgNPs. (**b**) TEM image of DCNC-AgNPs. (**c**) XPS spectra of CS-DCNC-AgNPs. (**d**) FTIR spectra of CS- DCNC-AgNPs.

**Figure 2:**
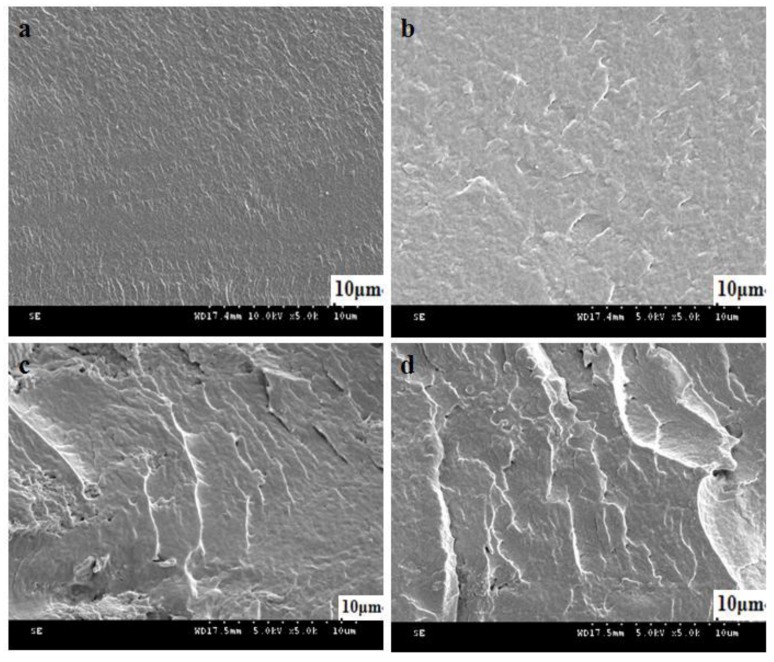
SEM image of the cross-section: (**a**) CS. (**b**) CS-DCNC-AgNPs (3%). (**c**) CS-DCNC-AgNPs (5%). (**d**) CS-DCNC-AgNPs (10%).

The UV-Vis absorption spectra (Figure 1a) of DCNC-AgNPs exhibited a single peak at about 414 nm, which clearly indicated the successful formation of AgNPs. Similar results were obtained by Biao et al. [26], who found that the absorption peak of AgNPs was 424 nm. The particle size ranged from 10 to 40 nm based on the observation of TEM (Figure 1b), which indicated that spherical AgNPs were present in the DCNC suspension. The XPS spectra showed the presence of the main elements in CS-DCNC-AgNPs, such as carbon (C1s), oxygen (O1s), nitrogen (N1s) and silver (Figure 1c). The characteristic peaks originated from CS were in good agreement with Arjunan et al. [21] and Li et al. [27]. The characteristic peak of CS-DCNC-AgNPs assigned to Ag (Ag3d) was observed at about 370.08 eV, which was evidence for the formation of AgNPs in DCNC suspension. According to a report by Arjunan et al. [21], Ag3d values of Ag-CS composites were between 368.2 eV and 374.2 eV. Our results were in good agreement with this report and confirmed the generation of AgNPs. All the absorbance bands of CS-DCNC-AgNPs were similar with those of pure CS, as proven by the FTIR spectra (Figure 1d). The main bands in the spectra of CS were located at around 1656 and 1590 cm^−1^; the first band resulted from –C=O stretching of the acetyl group, and the second band was the N–H bending vibrations of the amide and amine groups [18]. In the spectra of CS-DCNC-AgNPs, two characteristic shoulder peaks of CS assigned to the vibration of the –C=O and N–H were slightly shifted to 1648 and 1558 cm^−1^, with a significant decrease in intensity due to the addition of the DCNC-AgNPs. The results reflected the presence of interactions between Ag, O, and N atoms of these groups [28], and were in good agreement with previously reported results [26,27,28]. Biao et al. [26] reported that the reason of the peak shift was the interaction of nitrogen atoms of primary amine groups and amide groups with the AgNPs that reduced the carbonyl stretching –C=O and deformation vibration intensity of the N–H. Meanwhile, the intensity of N–H bending vibration bands at 1370 cm^−1^ decreased, which indicated the attachment of silver to nitrogen atoms [29]. Figure 2 represents the cross-section microstructure of CS-DCNC-AgNPs. It can be seen that, at a low concentration (3%), the CS-DCNC-AgNPs were evenly distributed in the polymeric matrix. However, dispersion of nanoparticles at the higher loading levels of DCNC-AgNPs (10%) was not uniform, and some agglomeration was observed. 

### 3.2. Mechanical Strength of CS-DCNC-AgNPs

The mechanical strength of CS with varying the DCNC-AgNPs concentration is summarized in Table 1. In the case of the dry samples, the tensile strength (TS) increased with increasing DCNC-AgNPs concentration. TS of CS-DCNC-AgNPs (10%) was 11% higher than that of CS, while the tensile modulus (TM) of CS-DCNC-AgNPs (10%) improved by 34% in comparison with CS. In contrast, the elongation at break (Eb) of CS-DCNC-AgNPs (10%) sharply decreased with increasing DCNC-AgNPs concentration, which decreased by nearly 114%. In case of the wet samples, the ultimate strength of wet CS was 0.9 MPa, CS-DCNC-AgNPs (10%) increased to 3.9 MPa with increasing DCNC-AgNPs concentration. Similarly, the TM value of wet samples also increased with increasing DCNC-AgNPs concentration. The Eb value of wet CS-DCNC-AgNPs (10%) was 30.5%, which is consistent with the same trend as in dry conditions. 

The increment in the TS values could be attributed to the nanocrystal-polymer interactions between DCNC and CS matrix due to similar polysaccharide structures of cellulose and chitosan [30], and also due to the reinforcing effect of the intermolecular hydrogen bonding [31]. All the samples of CS-DCNC-AgNPs showed higher values of TM than CS. CS-DCNC-AgNPs became more brittle, which was attributed to the increased stiffness in CS films by the addition of DCNC [7,20]. A significant low Eb value was observed, probably due to the incorporation of DCNC into CS matrix, which resulted in the strong interactions between them, and restricted the motion of matrix [32]. In the current work, we found that the incorporation of DCNC-AgNPs could improve the mechanical strength of CS. However, the effect of improvement was lower than that of earlier studies [7,20]. The explanation of this phenomenon was probably that the hydroxyl groups on the molecular chain of CNC were partially oxidized to aldehyde groups in the preparation of the DCNC, leading to the reduction in the relative crystallinity, thus reducing the mechanical performance of CNC [33]. From an earlier report by Wu et al. [30], CNC-CS nanocomposites have a similar architecture to the elastic tissue, and the TS of wet CNC-CS nanocomposites was in the range of 0.9–12.5 MPa, while the Eb was in the range of 81–23%. In another report by Cao et al. [34], the tensile strength of CNC-CS composite membranes was between 63.33 and 93.80 MPa. Our results matched the literature data well for human skin, whose stiffness was 0.1–2 MPa and 63% for failure stretch [30,35]. Taken together, the enhancement of mechanical strength by adding DCNC-AgNPs into CS is meaningful for application as a wound dressing.

### 3.3. Swelling Ratio of CS-DCNC-AgNPs

**Figure 3:**
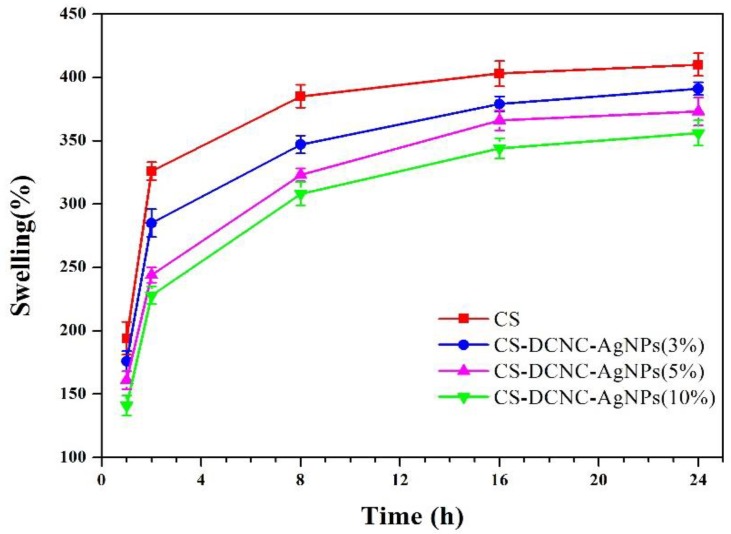
Swelling ratio of CS with varying DCNC-AgNPs concentration.

Wound dressings with good water barrier properties can delay the skin exudate, which can help the wound to form a moist healing environment, promote tissue regeneration, accelerate wound healing, allow gentle removal of the dressing without destroying the freshly formed tissue, and reduce the scar formation [36,37]. To study the swelling ratio of CS films with uniform thickness and different DCNC-AgNPs concentration, the films were immersed in water and the change in weight was observed at different time points, as shown in Figure 3. The maximum swelling ratios were 410%, 392%, 373%, and 356% for CS, CS-DCNC-AgNPs (3%), CS-DCNC-AgNPs (5%), and CS-DCNC-AgNPs (10%), respectively, after 24 h, which were dependent on the DCNC-AgNP concentration with CS films. All four kinds of samples had a rapid uptake of water during the first eight hours, after which the swelling ratio of each sample increased slowly until an equilibrium was reached. The most reasonable explanation for this behavior was that, as discussed previously, the DCNC acted as an inter-penetrated network within the matrix, and prevented the swelling of CS film. Moreover, the crystalline DCNC was less hydrophilic than CS, and formed strong filler-matrix interactions [7]. Another possible reason for the decrease in the water swelling capacity with an increasing DCNC-AgNPs concentration was the possible binding between the AgNPs and the hydroxyl and amine groups in the CS-DCNC chains that resulted in the partial crosslinking, and restricted the possibility of water absorption [28]. 

### 3.4. Antibacterial Activity of CS-DCNC-AgNPs 

**Figure 4:**
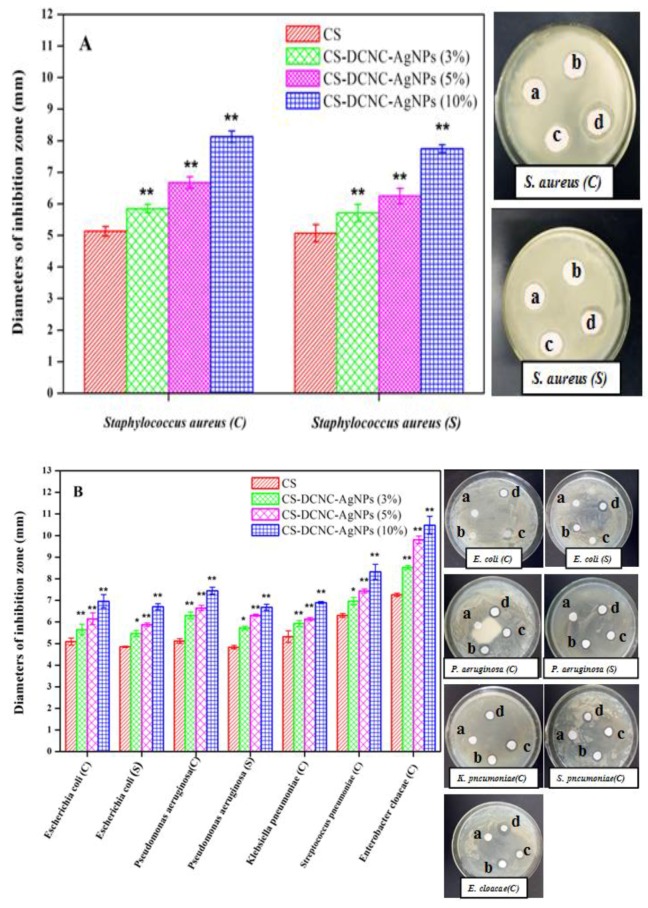

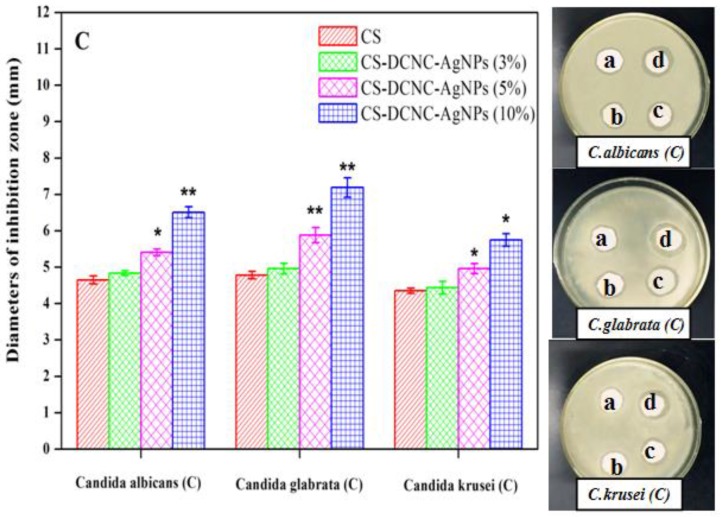
Antibacteria activity of CS-DCNC-AgNPs: (**A**) Gram-positive bacteria, (**B**) Gram-negative bacteria, (**C**) fungi. The meaning of the letter in the inset is: (**a**) CS, (**b**) CS-DCNC-AgNPs (3%), (**c**) CS-DCNC-AgNPs (5%), (**d**) CS-DCNC-AgNPs (10%).

The antibacterial activity of CS-DCNC-AgNPs was tested by disc diffusion assay. The change in the mean diameter of inhibition zones for CS with varying DCNC-AgNPs concentration is shown in Figure 4. The results revealed that CS-DCNC-AgNPs films inhibited the growth of the tested five gram-positive bacteria (Figure 4A), three gram-negative bacteria (Figure 4B), and three fungi (Figure 4C). Recently, the antibacterial effect of CS on fungi [38] and bacteria [39,40] have been extensively reported, and the antibacterial activity of CS incorporated in AgNPs has been demonstrated [41]. However, there is no research on the antibacterial activity of chitosan incorporated with DCNC-AgNPs. Our results showed that DCNC-AgNPs could improve the antibacterial effects of CS films, and the antibacterial activity of CS-DCNC-AgNPs was found to be directly related to the DCNC-AgNPs concentrations. The antibacterial activity of CS-DCNC-AgNPs (10%) against the Gram-positive bacteria: *Staphylococcus aureus*(C) and (S) with the diameters of inhibition zone were 8.13 and 7.75 mm, respectively. Similarly, CS-DCNC-AgNPs (10%) also inhibited the growth of Gram-negative bacteria. The diameters of inhibition zone were: *Escherichia coli*(C) (6.96 mm), *Escherichia coli*(S) (6.71 mm), *Pseudomonas aeruginosa*(C) (7.45 mm), *Pseudomonas aeruginosa*(S) (6.68 mm), *Klebsiella pneumoniae*(C) (6.90 mm), *Streptococcus pneumoniae*(C) (8.32 mm) and *Enterobacter cloacae*(C) (10.48 mm). Furthermore, the diameters of inhibition zone of three fungi were: *Candida albicans*(C) (6.41 mm), *Candida glabrata*(C) (7.19 mm) and *Candida krusei*(C) (5.89 mm). Taken together, CS-DCNC-AgNPs showed moderate antibacterial activity. 

The exact mechanism involved in the antibacterial activity of AgNPs is still unclear. However, earlier reports demonstrated that the antibacterial activity of AgNPs against the tested bacteria could be related to differences in their structure [28], and the lipid layer composition of the cell membrane [42]. Thiel et al. [43] reported that the cellular wall of gram-positive bacteria was wider than that of gram-negative bacteria. Zhang et al. [44] reported that the cell membrane of gram-positive bacteria consisted a thick peptidoglycan layer composed of a net structure with large numbers of pores and negatively charged teichoic acid, which allowed the cationic molecules to easily interact with the bacterium, and thereby, inhibited its growth. Das et al. [45] reported that the gram-negative bacteria had a thin outer membrane outside the peptidoglycan layer, due to the cell structure with complicated bilayer; the outer membrane could be regarded as a selective permeability barrier to protect the bacteria and to sustain its growth. 

The antibacterial effect and possible mechanisms of AgNPs involved in the deactivation of bacterial strains are known. However, little is known regarding the effects and mechanisms of AgNPs involved in the antifungal activity [46]. According to previous reports [47,48,49], fungi could interact with the AgNPs through several different mechanisms, including bio-accumulation, biosorption, micro-precipitation and chemical transformation. Most of the reports that referred to the antifungal activities of AgNPs were examined in relation to clinical isolates of *Candida albicans* [50,51]. In this work, we demonstrated that the growth of *Candida albicans*, *Candida glabrata*, *Candida krusei* were inhibited by the AgNPs. Our results also revealed the ability of AgNPs to impair the growth of fungi, and the effects varied depending on the concentration of AgNPs.

### 3.5. Cytotoxicity assay of CS-DCNC-AgNPs

**Figure 5:**
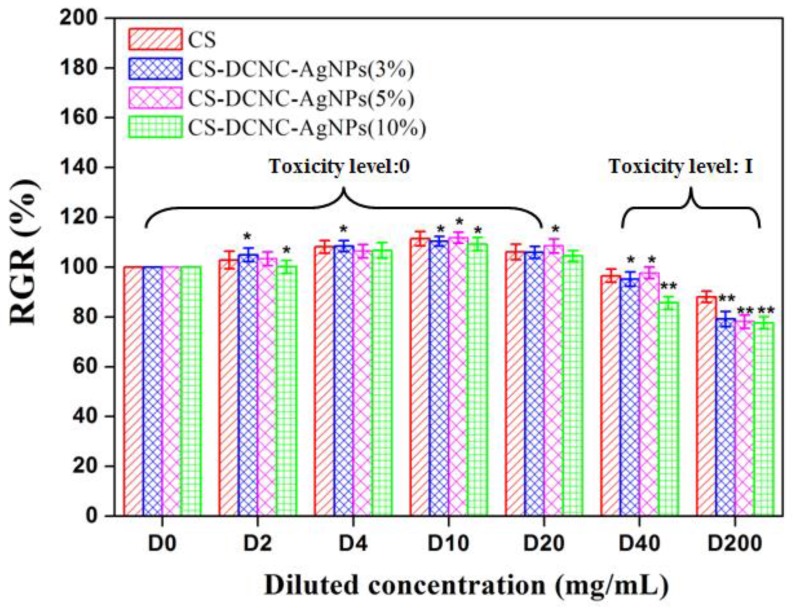
RGR values and cytotoxicity level of CS-DCNC-AgNPs: toxicity level 0 and I represent safe.

The cell RGR values and cytotoxicity potential of CS with varying DCNC-AgNPs concentrations are shown in Figure 5, at different dilutions. The cytotoxicity levels and RGR values of all groups were safe, and more than 75%, respectively. The cytotoxic effect of the CS-DCNC-AgNPs was found to increase with increasing DCNC-AgNPs concentration. This might be due to the higher DCNC-AgNPs content, which led to the cell damage by a cascade of processes like binding and reacting with proteins, phagocytosis, deposition, clearance, and translocation [52]. In fact, there was no report available regarding the cytotoxic effect of CS-DCNC-AgNPs on NIH3T3 cells; our results indicated that CS-DCNC-AgNPs possessed no cytotoxicity on the NIH3T3 cells. 

## 4. Conclusions

In the present work, we focused on the incorporation of AgNPs in DCNC by reducing the [Ag(NH_3_)_2_]^+^ complex to AgNPs (Ag^0^) that were loaded directly on the surface of DCNC. The hydrogen bonding between DCNC and CS undeniably improved the mechanical strength by crosslinking. On the other hand, the in-situ generated AgNPs significantly improved the antibacterial activity against gram-positive and gram-negative bacteria and fungi. Moreover, the cytotoxicity studies of CS-DCNC-AgNPs on NIH3T3 cells indicated that the generated conjugated complex was safe. Considering the previously mentioned merits, CS-DCNC-AgNPs seem to be a promising strategy for better antibacterial wound dressings, offering reduced toxicity and high mechanical strength. 

## Figures and Tables

**Table 1:** Mechanical strength of CS-DCNC-AgNPs.

Material	Tensile strength (MPa)		Tensile modulus (MPa)		Elongation at break (100%)	
	Dry	Wet	Dry	Wet	Dry	Wet
CS	48.5 ± 6.3	0.9 ± 0.2	1688 ± 153	0.7 ± 0.3	34.2 ± 5.3	65.2 ± 7.3
CS-DCNC-AgNPs (3%)	49.6 ± 3.5	1.5 ± 0.3	1931 ± 146	3.5 ± 0.4	22.4 ± 6.7	42.7 ± 9.4
CS-DCNC-AgNPs (5%)	54.2 ± 4.3	3.3 ± 0.6	2155 ± 182	5.9 ± 0.6	17.5 ± 4.6	34.3 ± 5.7
CS-DCNC-AgNPs (10%)	54.4 ± 5.4	3.9 ± 0.4	2263 ± 204	6.3 ± 0.5	15.2 ± 6.1	30.5 ± 4.8
